# Molecular Epidemiology and Antimicrobial Resistance of *Clostridioides difficile* in Hospitalized Patients From Mexico

**DOI:** 10.3389/fmicb.2021.787451

**Published:** 2022-03-10

**Authors:** Emmanuel Aguilar-Zamora, Bart C. Weimer, Roberto C. Torres, Alejandro Gómez-Delgado, Nayeli Ortiz-Olvera, Gerardo Aparicio-Ozores, Varenka J. Barbero-Becerra, Javier Torres, Margarita Camorlinga-Ponce

**Affiliations:** ^1^Unidad de Investigación Medica en Enfermedades Infecciosas y Parasitarias, UMAE Pediatría, CMN Siglo XXI, IMSS, México City, Mexico; ^2^Escuela Nacional de Ciencias Biológicas, Instituto Politécnico Nacional, México City, Mexico; ^3^Department of Population Health and Reproduction, School of Veterinary Medicine, 100K Pathogen Genome Project, University of California, Davis, Davis, CA, United States; ^4^Departamento de Gastroenterología, UMAE Hospital de Especialidades, Instituto Mexicano del Seguro Social, México City, Mexico; ^5^Departamento de Microbiología, Escuela Nacional de Ciencias Biológicas, Instituto Politécnico Nacional, México City, Mexico; ^6^Translational Research Unit, Medica Sur Clinic and Foundation, México City, Mexico

**Keywords:** *Clostridioides difficile*, antibiotic resistance, whole-genome sequencing, mutation, multilocus sequence typing, adults and children

## Abstract

*Clostridioides difficile* is a global public health problem, which is a primary cause of antibiotic-associated diarrhea in humans. The emergence of hypervirulent and antibiotic-resistant strains is associated with the increased incidence and severity of the disease. There are limited studies on genomic characterization of *C. difficile* in Latin America. We aimed to learn about the molecular epidemiology and antimicrobial resistance in *C. difficile* strains from adults and children in hospitals of México. We studied 94 *C. difficile* isolates from seven hospitals in Mexico City from 2014 to 2018. Whole-genome sequencing (WGS) was used to determine the genotype and examine the toxigenic profiles. Susceptibility to antibiotics was determined by *E*-test. Multilocus sequence typing (MLST) was used to determine allelic profiles. Results identified 20 different sequence types (ST) in the 94 isolates, mostly clade 2 and clade 1. ST1 was predominant in isolates from adult and children. Toxigenic strains comprised 87.2% of the isolates that were combinations of *tcdAB* and *cdtAB* (*tcdA+/tcdB+/cdtA+/cdtB+*, followed by *tcdA+/tcdB+/cdtA−/cdtB−*, *tcdA*−/*tcdB+/cdtA−/ cdtB−*, and *tcdA−*/*tcdB−/cdtA+/cdtB+*). Toxin profiles were more diverse in isolates from children. All 94 isolates were susceptible to metronidazole and vancomycin, whereas a considerable number of isolates were resistant to clindamycin, fluroquinolones, rifampicin, meropenem, and linezolid. Multidrug-resistant isolates (≥3 antibiotics) comprised 65% of the isolates. The correlation between resistant genotypes and phenotypes was evaluated by the kappa test. Mutations in *rpoB* and *rpoC* showed moderate concordance with resistance to rifampicin and mutations in *fusA* substantial concordance with fusidic acid resistance. *cfrE*, a gene recently described in one Mexican isolate, was present in 65% of strains linezolid resistant, all ST1 organisms. WGS is a powerful tool to genotype and characterize virulence and antibiotic susceptibility patterns.

## Introduction

*Clostridioides* (*Clostridium*) *difficile* is a spore-forming, gram-positive, and anaerobic bacillus found in the environment and in the intestinal tract of animals and humans. In humans, the infection is the leading cause of antibiotic-associated diarrhea and of a wide range of gastrointestinal syndromes (Knight et al., 2015; Turner and Anderson, 2020). The molecular epidemiology of *C. difficile* infection (CDI) has shown that the bacterial genome and the disease have become very variable in the last decades. The incidence of CDI markedly increased worldwide at the end of the twentieth century (Czepiel et al., 2019), which was associated with the rapid spread of the hypervirulent strain NAP1/B1/027/ST01 [North American Pulse field type 1/restriction endonuclease analysis type BI/ribotype 027/multilocus sequence typing (MLST)] (Krutova et al., 2018; Lv et al., 2019; Guerrero-Araya et al., 2020). In addition, CDI cases attributed to other ribotypes such as RT078, RT001, RT018, and RT126 are emerging in Europe (Couturier et al., 2018), and, currently, CDI is the most frequently identified health care–associated infection in the United States (Guh and Kutty, 2018).

A number of major factors contribute to the virulence of *C. difficile* including the production of toxin A (TcdA) and toxin B (TcdB), which are monoglycosyltransferases that disrupt the gut epithelium (Monot et al., 2015), as well as other factors that participate in colonization like adhesins, pili, and flagella (Janoir, 2016). The toxins are encoded by *tcdA* and *tcdB* genes that are situated in the pathogenicity locus (PaLoc) and are implicated in progression and severity of CDI (Monot et al., 2015). In addition, some *C. difficile* strains express an ADP-ribosylating toxin named *C. difficile* transferase (CDT) that modifies actin and is encoded by the genes *cdtA* and *cdtB* located in the CdTLoc locus (Gerding et al., 2014).

The use of antibiotics induces transmission of *C. difficile*. Many antibiotics are associated with CDI; ampicillin, amoxicillin, cephalosporins, clindamycin, and fluoroquinolones continue to be associated with the highest risk for CDI (Spigaglia, 2016; Banawas, 2018). Understanding the mechanisms of resistance of *C. difficile* is a key issue in the strategy to control spread of CDI (Peng et al., 2017). Resistance to tetracycline, chloramphenicol, and linezolid is less frequently associated with CDI with differences between countries (Sholeh et al., 2020).

*C. difficile* has a versatile genome content, with a wide range of mobile elements, many of them encoding for antibiotic resistance (Spigaglia, 2016). Transposons that confer resistance to lincomycin and streptogramin B (Tn5398 and Tn6194) (Mullany et al., 2015), to chloramphenicol (Tn4453a and Tn4453b) (Mullany et al., 2015; Peng et al., 2017), to erythromycin (Tn5398), or to tetracycline (Tn916-like and Tn5397) exist in various isolates (Spigaglia et al., 2018). Recently, a *cfr*-like gene named *cfrE* was described in a Mexican *C. difficile* isolate and appears to be associated with resistance to phenicols, lincosamides, oxazolidinones, pleuromutilins, and streptogramin A (Stojković et al., 2020). Mutations in *gyrA* and *gyrB* are associated with resistance to fluoroquinolones, whereas missense mutations in the *rpoB* gene confer resistance to rifaximin and rifampicin (Peng et al., 2017). Currently, standard CDI therapies include metronidazole and vancomycin as the first choice for primary mild and severe CDI, respectively (Spigaglia et al., 2010); however, some studies have recently reported resistance or reduced susceptibility to metronidazole and vancomycin (Chahine, 2018). At present, rifaximin and fidaxomicin are recommended as the antibiotic of choice for relapsing or recurrent CDI (Spigaglia, 2016).

Whole-genome sequencing (WGS) is a tool that allows studies on the diversity, plasticity, and population structure of the *C. difficile* genomes and helps understand the complexity of CDI management including antibiotic resistance (Knight et al., 2015; Saldanha et al., 2020) and toxin variants (Li et al., 2020). It also facilitates understanding the *C. difficile* epidemiology, providing information on the spread, emergence, and detection of strains with increased virulence using genome differences (Knetsch et al., 2013). MLST analyses of housekeeping genes are accepted as a reliable tool for routine typing of CDI; it provides highly reproducible and easy to interpret results as compared to other typing methods (Kamboj et al., 2021), although it is not the best choice for epidemiological studies, where genome-based analyses are currently applied, including core genome MLST (cgMLST) (Bletz et al., 2018; Janezic and Rupnik, 2019).

Although CDI is an important cause of hospital-acquired diarrhea and colitis in Latin America (Muñoz et al., 2018; Guerrero-Araya et al., 2020), little is known about antibiotic resistance and molecular epidemiology of *C. difficile* in this region. In recent years, studies in Mexico have focused mainly on molecular typing of *C. difficile* strains, particularly on the identification of the hypervirulent strain RT027 using PCR ribotyping (Camacho-Ortiz et al., 2015; Martínez-Meléndez et al., 2018). The aim of this study was to examine the molecular epidemiology of *C. difficile* strains isolated from patients at hospitals in Mexico. WGS was used to genotype, determining the genotype of antibiotic resistance, and the profile of toxins in *C. difficile* strains isolated from adults and children.

## Materials and Methods

### Patients and Isolation of *Clostridioides difficile*

A total of 94 *C. difficile* strains were isolated from stool samples of patients with hospital acquired diarrhea; of these, 31 were isolated from children and 63 from adults. Patients were recruited from seven hospitals (one pediatric and six general hospitals) in Mexico City between 2014 and 2018. Hospitals requested *C. difficile* culture from clinically suspected cases, and, from a collection of 160 isolated strains, we selected 63 strains from adults and 31 from children for WGS; selected isolates were those that were sequentially recovered from our frozen collection, and the total number was limited by the available funds for sequencing.

To isolate C. *difficile*, stool samples were treated with 96% ethanol at room temperature for 50 min followed by centrifugation at 4,000 rpm for 10 min. The cell pellets were inoculated onto taurocholate–cefoxitin–cycloserine fructose agar plates and incubated at 37^°^C for 5 days in an anaerobic jar with an atmosphere containing 85% N_2_, 5% H_2_, and 10% CO_2_ that was generated using the Anoxomat system (MART Microbiology B.V., The Netherlands). *C. difficile* isolates were identified by their characteristic colony morphology, gram stain, colony fluorescence, and odor. Identification was confirmed using the Vitek MS combined with detection of the *tpi* gene by PCR using primers and conditions previously reported (Lemee et al., 2004). All isolates were frozen at −70^°^C in Brucella broth medium supplemented with 10% glycerol for subsequent analysis.

### Ethical Considerations

The study protocol was approved by the ethical committee of Instituto Mexicano del Seguro Social, and all adult participants or guardians of children were informed about the study and asked to sign a consent letter.

### Antimicrobial Susceptibility Assay

The antimicrobial susceptibility of *C. difficile* to clindamycin, ciprofloxacin, levofloxacin, moxifloxacin, linezolid, metronidazole, and vancomycin was determined by the Epsilometric method (*E*-test) on pre-reduced Brucella agar (BBL BD, United States) containing 5% of defibrinated sheep blood, vitamin K (1 μg/ml), and hemin (5 μg/ml). *E*-test strips (Liofilchem, Italy) were placed on the plate and incubated at 37^°^C for 48 h, in anaerobic conditions (85%N_2_, 5%H_2_, and 10% CO_2_). The minimal inhibitory concentration (MIC) was defined by the point of intersection of the inhibitory zone with the strip, whereas susceptibility to tetracycline, rifampicin, fusidic acid, and meropenem was determined by the agar dilution method. Brucella Agar (BBL BD, United States) was also used but mixed with the antimicrobial agent solution, following the guidelines by the Clinical and Laboratory Standards Institute (CLSI) (Clinical and Laboratory Standards Institute, 2018) and the guidelines by the European Committee on Antimicrobial Susceptibility Testing (EUCAST) (v.2.0).^1^ Cutoff values were adopted from the CLSI and EUCAST guidelines for anaerobic bacteria; the breakpoints used to define resistance were as follows: >16 μg/ml for rifampicin, meropenem, and tetracycline; >4 μg/ml for linezolid; 0.5 μg/ml for fusidic acid; >32 μg/ml for metronidazole; >4 for vancomycin; and >8 μg/ml for moxifloxacin, levofloxacin, clindamycin, and ciprofloxacin.

### DNA Extraction and Amplification of *Clostridioides difficile* Housekeeping and Toxins Genes

Genomic DNA was prepared from Brucella broth culture of *C. difficile* strains grown under anaerobic conditions at 37^°^C for 48 h. The culture was harvested by centrifugation (14,000 rpm for 2 min); washed in sterile phosphate buffered solution (PBS); resuspended in 180 μl of lysis buffer comprising 20 mM Tris-HCl, pH 8.0, 2 mM EDTA, and lysozyme (20 mg/ml); and incubated for 30 min at 37^°^C. DNA was extracted using a DNeasy^®^ Kit (Qiagen, Hilden Germany) according to the manufacturer’s instructions (Aguayo et al., 2015). The housekeeping genes *tpi* and *tcdA* (toxin A), *tcdB* (toxin B), and cdtA/*cdtB* (binary toxin) were amplified by PCR as previously described (Lemee et al., 2004; Persson et al., 2008). Positive controls consisted of DNA template from *C. difficile* ATCC 630 and *C. difficile* ATCC 9689.

### Whole-Genome Sequencing and Phylogenomic Analysis

Isolates were sequenced at the University of California, Davis (United States) within the 100K Pathogen Genome Project (Weimer, 2017). WGS of the 94 Mexican *C. difficile* strains was done using PE150 on a HiSeq 2500 platform (Illumina Inc., San Diego, CA, United States) (Miller et al., 2019). Genomes were assembled *de novo* with the Shovill pipeline^2^ (Trinetta et al., 2020) using default settings, and the quality of assemblies was assessed using QUAST v.5.0.0^3^ ; genomes with contamination or low coverage (<33×) were discarded. The contigs were annotated using rapid annotation pipeline Prokka v.1.13^4^ (Seemann, 2014). All genome sequences were deposited in the NCBI as part of the 100K Pathogen Genome Project BioProject under the accession number PRJNA203445; Supplementary Table 1 describes the accession number for each genome sequence.

Genomes were screened for the *tcdA*, *tcdB*, and *cdtA*/*cdtB* and other virulence genes using the Virulence Factors Database from Resfinder^5^ (Hu et al., 2020) as well as the annotation provided by Prokka (see text footnote 4) (Seemann, 2014).

Raw sequence data files of the isolates were uploaded to EnteroBase web--based platform^6^ for core genome analysis. Analysis includes pre-processing, trimming, assembly, post-correction, and filtering, and the output is a FASTA file used for analysis including MLST on different levels (Zhou et al., 2020). EnteroBase includes up to now 23,632 C. *difficile* genomes. We choose the cgMLST scheme, which contains a subset of 2,556 loci, to analyze our 94 strains. Each genome has been assigned to hierarchical sets of single-linkage clusters by cgMLST distances. This hierarchical clustering is used to identify and name populations of *C. difficile* for epidemiological studies (Frentrup et al., 2020). The phylogenetic cluster analysis was plotted by neighbor joining tree that was visualized using the R packages ggplot2 (v3.0.0) (Wickham, 2016) and ggtree (v2.4.1) (Yu et al., 2017).

### Multi-Locus Sequence Typing Analysis

Multi-Locus Sequence Typing (MLST) of all isolates was performed using seven housekeeping genes as previously described (Griffiths et al., 2010). The assignation of *C. difficile* sequence type (ST) and clades was done according to PubMLST database using MLST v.2.10.^7^ To show the genetic diversity of the MLST results, a maximum-likelihood tree was generated with MUSCLE-aligned concatenated allele sequences using PhyML v3.0 with a Hasegawa–Kishino–Yano evolutionary model and 1,000 random bootstrap replicates (Edgar, 2004; Guindon et al., 2009).

### Bioinformatic Analysis of Antibiotic Resistance

We identified antimicrobial resistance (ARG) genes by screening contigs with ResFinder (Bortolaia et al., 2020) and CARD^8^ using ABRicate version 1.0.1^9^ (Seemann, 2020). The HMMER program v.2.1.1 was used to build Hidden Markov Model to search for *cfrE* gene. Analysis of previously reported substitutions related to antibiotic resistance in *gyrA*, *gyrB*, *rpoB*, *rpoC*, *fusA*, *pbp2*, and *pbp3* (Isidro et al., 2018) was retrieved using Snippy v.4.6.0,^10^ mapping the assembled *C. difficile* Mexican genomes against *C. difficile* 630 reference genome (sequence accession number AM180355.1). Analysis of single nucleotide polymorphisms (SNPs) within antibiotic resistance genes was also done using Snippy (v.4.6.0) BWA. MEM 1.2.0 (Li and Durbin, 2010; see text footnote 10).

To determine the relation between *C. difficile* clades and antimicrobial resistance, a phylogenetic tree was constructed with whole-genome sequences using virtual genome fingerprint with VAMPhyRE software^11^ with a probe set of 13 mers, allowing one mismatch and using a threshold of 17 nucleotides.

### Statistical Analysis

The frequency of resistance to one or more antibiotics among the study populations was analyzed, and their 95% confidence intervals were estimated. *Z*-test for comparison for two proportions was used to evaluate the frequency of differences in antibiotic resistance, toxin profile, and MLST clades between both children and adult isolates. All statistical analyses were performed in OMS-Epidata version 4.2 (2016).^12^ The agreement between antibiotic resistance phenotype and genotype was tested using a Cohen’s kappa statistics. A kappa coefficient value of <0.4, 0.4–0.6, 0.61–0.8, and 0.81–1.0 indicated low, moderate, substantial, and perfect agreement, respectively (Liou et al., 2011). In addition, a *p*-value <0.05 was considered as statistically significant in the above tests.

## Results

### *Clostridioides difficile* Isolates

A total of 94 *C. difficile* strains were obtained from patients from seven hospitals in Mexico City. Thirty-one isolates were from children (mean age, 7.4 ± 5.8; 12 females and 19 males), whereas 63 were isolated from adults (mean age, 58.9 ± 17.3 years; 38 females and 24 males).

### Toxin Profile

**FIGURE 1:**
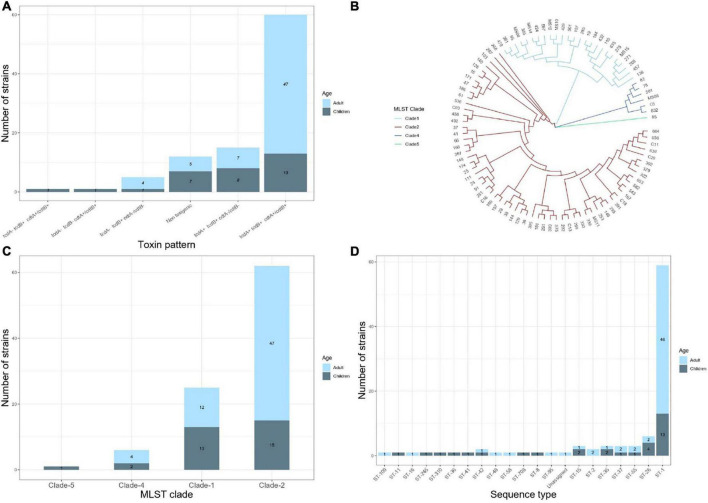
Results of analysis of the whole genome sequences of 94 *Clostridiodes difficile* isolates from 63 adults and 31 children. **(A)** Distribution of toxins profile of the isolates. **(B)** Vamphyre phylogenetic circular tree based on MLST analysis to illustrate the genetic relationships between 20 STs that involve four clades of *C. difficile.* Each color corresponds to clades (Clade 1 blue, Clade 2 red, Clade 4 dark blue, Clade 5 green). **(C)** Number of *C. difficile* strains per MLST clade. **(D)** Number of STs identified by MLST in the studied isolates.

The presence of *tcdA, tcdB, cdtA*, and *cdtB* genes was examined using PCR initially and confirmed with the WGS in all strains. Figure 1A depicts the toxins profiles. A toxigenic profile (those containing at least one toxin gene) was found in 82 (87.2%) of the isolates, whereas 12 (12.7%) isolates contained no toxin genes and were considered to be non-toxigenic. Among the 82 toxigenic strains (24 from children and 58 from adults), the most frequent toxin profile was *tcdA+/tcdB+/cdtA+/cdtB+*. This profile was significantly different between children (41.9%) and adults (81%) (*p* = 0.02). Conversely, the toxin profile *tcdA+/tcdB+/cdtB−/cdtB−* was more frequent in children (25.8%) than adults (12%) (*p* = 0.067). The *tcdA−*/*tcdB+/cdtA−/cdtB− was* present in five *C. difficile* isolates (four from adults and one from children). One *C. difficile* isolate from children contained only the *cdtB+* gene, and another isolate from a child contained the unusual *tcdA−/tcdB+/cdtA+/cdtB+* combination of toxin genes.

### Multi-Locus Sequence Typing

The MLST relationships of the 94 *C. difficile* isolates formed four clades and 20 different ST groups (Figure 1B). Clade 2 was the most frequent, although its frequency was higher in adults (74.6%) than in children (48.3%) (*p* = 0.012). In contrast, clade 1 was more frequent in children (41.9%) than in adults (19%) (*p* = 0.018) (Figure 1C). Four isolates from adults and two from children were in clade 4 and only one isolate from a child belonged to clade 5. ST1 (NAP1/027) was the most common type, accounting for 63% of all the isolates, corresponding to 45/63 (71.4%) and 13/31 (41.9%) of the strains isolated from adult and children, respectively (Figure 1D). The remaining STs were represented by one or two isolates, except ST26 (RT039/140, clade 1) identified in four isolates from children. Of note, the following STs were identified only in isolates from children, ST8 (RT002, clade 1), ST11 (RT078, clade 5), ST36 (RT011, clade 1), ST41 (RT244, clade 2), ST310 (clade 4), and ST708. Whereas ST2 (RT014/020/076/220, clade 1), ST16 (RT050, clade 1), ST48 (clade 1), ST58 (clade 1), ST95 (clade 2), and ST109 (clade 4) were identified only in adult isolates.

### Antibiotic Resistance

Antimicrobial susceptibility testing of the 94 isolates was done using 11 antimicrobial agents (Table 1). The pattern of resistance was similar in both *C. difficile* isolates from children and adults except for levofloxacin, rifampicin, and linezolid where resistance was significantly lower in pediatric isolates (Table 1). Isolates from children were resistant to fluoroquinolones at 100% for both ciprofloxacin and moxifloxacin and 77.4% for levofloxacin. Similarly, isolates from adult were largely resistant to fluoroquinolones with moxifloxacin (90.5%), ciprofloxacin (98.4%), and levofloxacin (95.2%). Resistance to tetracycline and fusidic acid was common among isolates from children and adults.

**TABLE 1:** Distribution of resistance pattern among Mexican *C. difficile* strains.

Resistance pattern	Number of strains				

	Adults		Children		
	*n* = 63		*n* = 31		
Antibiotic	No.	%(95CI)	No.	%(95CI)	*p*-value
Clindamycin (CLIN)	56	88.8 (78.4–95.4)	27	87.1 (70.1–96.4)	0.799
Levofloxacin (LEV)	60	95.2 (86.7–99.0)	24	77.4 (58.9–90.4)	0.008
Ciprofloxacin (CIPRO)	62	98.4 (91.4–99.9)	31	100 (–)	0.958
Moxifloxacin (MOX)	57	90.4 (80.4–96.4)	31	100 (–)	0.749
Rifampicin (RIF)	54	85.7 (74.6–93.2)	20	64.5 (45.3–80.7)	0.018
Linezolid (LIN)	43	68.2 (55.3–79.4)	14	45.1 (27.3–63.9)	0.031
Meropenem (MER)	42	66.6 (53.6–78.0)	23	74.1 (55.3–88.1)	0.458
Fusidic Acid (FUS)	6	9.5 (3.5–19.5)	5	16.1 (5.4–33.7)	0.349
Tetracycline (TET)	6	9.5 (3.5–19.5)	1	3.2 (0.08–16.7)	0.274
Vancomycin (VAN)		0		0	
Metronidazole (MET)		0		0	
**Multiple resistance**					
CLIN, CIPRO, MERO	41	65.0 (52.0–76.6)	20	64.5 (45.3–80.7)	0.957
CLIN,LEV,CIPRO,RIF, LIN	28	44.4 (31.9–57.5)	12	38.7 (21.8–57.8)	0.597
CLIN,LEV,CIPRO,RIF, MER	38	60.3 (47.2–72.4)	14	45.1 (27.3–63.9)	0.165
CLIN,LEV,CIPRO,RIF, MER, TET	4	6.3 (1.7–15.4)		0	

**FIGURE 2:**
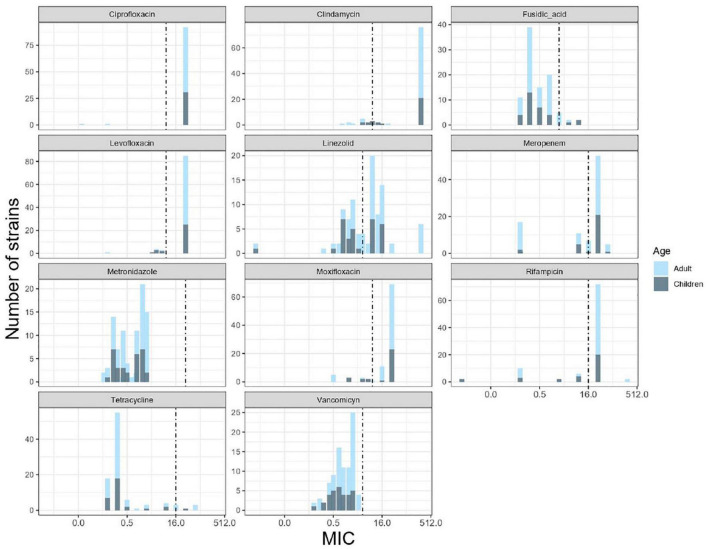
Minimum inhibitory concentration (MIC) distributions for 11 antibiotics against 94 *C. difficile* isolates of children and adults. The graphs show the number of isolates across a range of MIC values. Black dashed lines indicate the clinical breakpoints according to the European Committee for Antimicrobial Susceptibility Testing (EUCAST).

Multiple resistance pattern to clindamycin, ciprofloxacin, and meropenem was frequent in *C. difficile* isolates from both children (64.5%) and adults (65%). Resistance to five antibiotics was common among isolates from adults (44%) and children (40%). Four isolates from adults contained resistance to six antibiotics (6.3%). The distribution of MIC values for each antibiotic is shown in Figure 2. High MIC values were observed for ciprofloxacin, levofloxacin, moxifloxacin clindamycin, meropenem, and rifampicin, with MIC values of 1.5–32 μg/ml for fluoroquinolones and 1.5–256 μg/ml for clindamycin. Among the other antibiotics tested, fusidic acid and tetracycline demonstrated a wide distribution of MIC values ranging from 0.125 to 8 μg/ml and from 0.125 to 64 μg/ml, respectively. All 94 *C. difficile* isolates tested were found susceptible to vancomycin and metronidazole (MICs 0.125–3 μg/ml and 0.094–2 μg/ml, respectively). Regarding the ST, over 95% of the ST1 isolates were resistant to ciprofloxacin, clindamycin, levofloxacin, moxifloxacin, and rifampin, whereas 74% were resistant to linezolid and meropenem, 41.3% to fusidic acid, and 6.8% to tetracycline.

### Molecular Analysis of Mechanisms of Resistance

**FIGURE 3:**
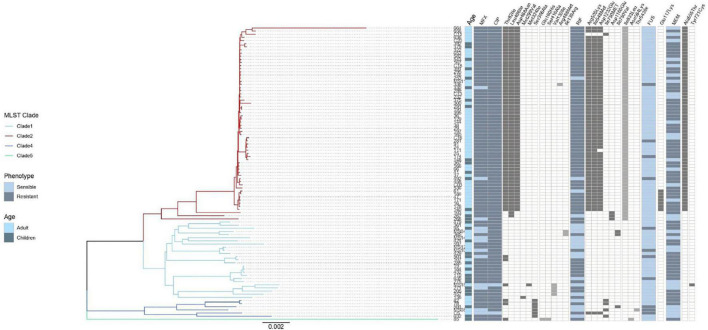
Result of genomic analysis of 94 *C. difficile* strains from Mexican patients and presence of resistance associated mutations. A phylogenetic tree based on whole genome sequences was constructed using virtual hybridization analysis (VAMPhyRE) and correlated with clades, patient’s age and with the presence of antibiotic resistance mutations. Presence of mutations is indicated by gray rectangles and absence by white rectangles. The sensitive and resistant phenotype are denoted by gray or blue rectangles, respectively, MFX (Moxifloxacin), CIP (Ciprofloxacin), RIF (Rifampicin) FUS (Fusidic acid), MEM (Meropenem). The presence and absence of mutations are denoted by black and white rectangles, respectively.

No correlation was observed between the presence of mutations in antimicrobial resistant genes and phenotypic resistance in the *C. difficile* strains (Table 2), except in three cases. Three mutations in *rpoB* and one in *rpoC* presented a moderate agreement with resistance to rifampicin, whereas the mutation Glu117Lys in *fusA* showed a substantial agreement with resistance to fusidic acid. High levels of rifampicin resistance (MIC above 16.0 μg/ml) could be due, in part, to multiple substitutions in RNA polymerase sub-unit B *rpoB* (Arg505Lys, Ile548Met, Ile750Met/Val, Asp1160Glu, and Asp1232Glu) that were detected in 70 *C. difficile* isolates from children and adults (Figure 3). Furthermore, substitution of Ile833Leu in *rpoC* was also frequent and probably also affecting susceptibility to rifampicin (kappa coefficient of 0.479).

**TABLE 2:** Concordance between genotypic and phenotypic drug resistance.

Gene	Antibiotic	Mutation	Kappa Coefficiency (95%CI)
		Thr82Ile	0.2937 [0.0933 – 0.4940]
		Leu406Ile	0.3491 [0.1654 – 0.5328]
*gyrA*	Moxifloxacin	Asp468Asn	0.3215 [0.1444 – 0.4985]
		Met299Val	0.0034 [−0.0035 – 0.0104]
		Met324Ile	−0.0215 [−0.0637 – 0.0208]
		Thr82Ile	0.0257 [−0.0721 – 0.1236]
		Leu406Ile	0.0773 [−0.0252 – 0.1798]
*gyrA*	Ciprofloxacin	Asp468Asn	0.0707 [−0.0236 – 0.1650]
		Met299Val	0.0005 [−0.0007 – 0.0016]
		Met324Ile	0.0215 [−0.0639 – 0.0209]
		Ser366Ala	−0.0305 [−0.0919 – 0.0309]
		Gln160His	0.0034 [−0.0035 – 0.0104]
*gyrB*	Moxifloxacin	Ser416Ala	0.0034 [−0.0035 – 0.0104]
		Val130Ile	−0.0370 [−0.0978 – 0.0237]
		Arg488Met	0.0034 [−0.0035 – 0.0104]
		Ile139Arg	−0.0182 [−0.0608 – 0.0244]
		Ser366Ala	−0.0202 [−0.0626 – 0.0221]
		Gln160His	0.0005 [−0.0007 – 0.0016]
*gyrB*	Ciprofloxacin	Ser416Ala	0.0005 [−0.0007 – 0.0016]
		Val130Ile	−0.0208 [−0.0631 – 0.0216]
		Arg488Met	0.0005 [−0.0007 – 0.0016]
		Ile139Arg	0.0009 [−0.0009 – 0.0028]
		Arg505Lys	0.4764 [0.2961 – 0.6567]
		Ile548Met	0.4764 [0.2961 – 0.6567]
*rpoB*	Rifampicin	Asp1232Glu	0.4592 [0.2908 – 0.6670]
		Ile750Met	0.0544 [−0.1297 – 0.0209]
		Asp1160Glu	−0.01 [−0.0549 – 0.0348]
		Ile750Val	−0.0377 [−0.0986 – 0.0232]
		Ile833Leu	0.4789 [0.2908 – 0.6670]
*rpoC*	Rifampicin	Asn564Lys	0.0058 [−0.0058 – 0.0174]
		Thr543Ile	−0.0214 [−0.0636 – 0.0207]
fusA	Fusidic Acid	Glu117Lys	0.7176 [0.5245 – 0.9107]
*pbp2*	Meropenem	Ala555Thr	0.1039 [−0.0991 – 0.3068]
*pbo3*	Meropenem	Tyr721Cys	−0.0214 [−0.0634 – 0.0206]

The substitutions Thr82Ile, Leu406Ile, Asp468Asn, Met299Val, and Met324Ile in *gyrA* were detected in 68 *C. difficile* strains (Figure 3); however, there was disagreement with phenotypic resistance for these substitutions. In addition, the substitutions Ser366Ala, Gln160His, Ser416Ala, Val130Ile, Arg488Met, and Ile139Arg in *gyrB* were found in 14 C. *difficile* isolates. These substitutions were not associated with resistance in the measured phenotype.

Seven ST1 isolates contained a SNP in *fusA* (Glu117Lys) with very high linkage with phenotypic resistance as determined using a kappa coefficient of 0.7176. Finally, 59 strains contained the A555T substitution in penicillin-binding protein 2 (*pbp2*) but only one had a Y721S substitution in *pbp3*. This substitution has been linked with an increase in meropenem resistance; however, the kappa coefficient showed a very low correlation with phenotype resistance in both cases (see Table 2).

**FIGURE 4:**
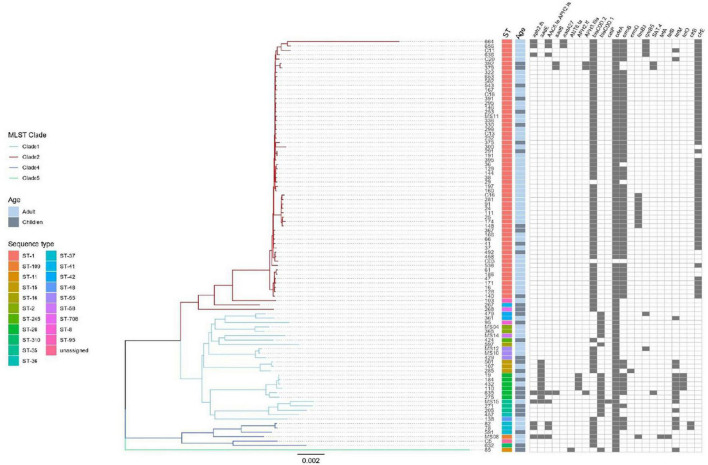
Result of genomic analysis of 94 *C. difficile* strains from Mexican patients and presence of resistance associated genes. Phylogenetic tree based on whole genome sequences was constructed using virtual hybridization analysis (VAMPhyRE) and correlated with clades, STs, patient’s age and with the presence of antibiotic resistance genes. Presence is indicated by gray rectangles and absence by white rectangles.

The genomic analysis showed than all 94 *C. difficile* isolates contained the multidrug and toxic compound extrusion (MATE) multidrug efflux transporter *cdeA* (Figure 4), whereas 66 isolates (70.2%) were positive for the methyltransferase *ermB* and only one for *ermQ*. In addition, two linezolid resistant strains (2.1%) also carried the rRNA methyltransferase *cfrB*; whereas the recently described *cfrE* gene was identified in 37 of 57 (64.9%) linezolid resistant strains, showing a perfect agreement with phenotype (kappa coefficient of 0.85). Moreover, a diverse collection of tetracycline resistance genes was identified with a varied distribution in MLST clades, and *tetM, tetO, tetB*, and *tetA* were found in 18.1, 4.3, 1.1, and 1.1% of the isolates, respectively. Components of an aminoglycoside-streptothricin resistance cassette (*ant6-sat4-alph-III*) were identified in 11.7% of the isolates. Three genes encoding putative aminoglycoside-modifying enzymes, termed *aadE* (aminoglycoside 6-adenylytrasnfesare), *aadA27* (aminoglycoside (3′′) (9) adenylyltransferase), and *aac(6′)-Ie-aph(2′′)-Ia* [bifunctional aminoglycoside N-acetyltransferase AAC(6′)-Ie/aminoglycoside O-phosphotransferase APH(2′′)-Ia] were found in 11.7, 2.1, and 8.5% of the isolates, respectively. The chloramphenicol resistance gene *catP* was present in one only (1.1%) strain. Finally, β-lactamase *blaCCD1* and *blaCDD2* were found in 19.1% (*n* = 18) and 79.8% (*n* = 75) of the 94 genomes, respectively (Figure 3).

### Whole-Genome Analyses and Correlation With Resistance, Toxins Profile, and Epidemiologic Variables

A phylogenetic analysis was done with the whole genomes using VAMPhyRE (Figure 4) that showed clade 2 with a reduced diversity in ST and resistant genes as compared with the other clades. The genome analysis clearly separated clusters within clades 1 and 4 and even within clade 2 where the analysis clearly separated three isolates (103, 267, and 268) from the other strains. Most strains in clade 2 were closely related and presented the *blaCDD2*, *cdeA*, and *emB* resistance-associated genes, as well as *cfrE*, a gene that has not been reported in other *C. difficile* populations. The other resistance-associated genes were variable present within clusters of clades 1, 4, and 5. Mutations associated to antibiotic resistance were more common among clade 2 isolates (Figure 3), and some mutations (Ser366Ala) were found only in clade 4 isolates.

**FIGURE 5:**
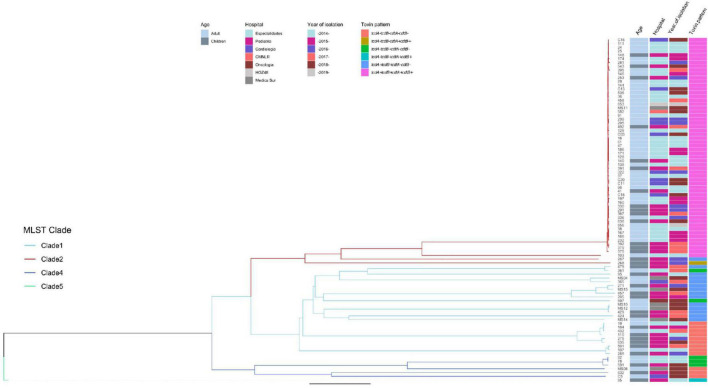
Neighbor Joining phylogenetic tree based on the cgMLST allelic profiles determined by EnteroBase and its relationship with other epidemiological variables. Columns on the right describe: Strain number, age of the patient, hospital, year of isolation and toxins profile are shown in relation to the cgMLST and MLST clades. MLST Clades are colored in the phylogenetic tree, Clade 1 blue, Clade 2 red, Clade 4 dark blue, and Clade 5 green.

Genomes were submitted to EnteroBase for a cgMLST analysis (Figure 5). The study grouped strains following the pattern of the clades, although the extended core analysis resulted in a more detailed clustering within clades, similar to the virtual hybridization assay. Genomic diversity was higher within strains of clades 1, 3, and 4, as compared to clade 2 where genomes seem to be more related. Still, within clade 2, there were clusters grouping isolates by age, hospital, and year of isolation (adult strains 38, 167, 166, and 232 during 2014–2015; and children strains 392 and 379 during 2017). All strains from clade 2 were *tcdA+/tcdB+/cdtA+/cdtB*+, except for two that clearly separated from most other isolates 267 (*tcdA+/tcdB+/cdtB−/cdtB−*) and 268 (*tcdA−/tcdB−/cdtA+/cdtB+*). Distances between genomes of clade 1 were relatively large, with few clusters like adult strains MS10 and MS12 isolated in 2018 or adult strains 19 and 432 together with children strain 184 recovered during 2014, 2015, and 2016. Toxins profiles in clade 1 were mostly *tcdA+/tcdB+/cdtB−/cdtB−* or *tcdA−/tcdB−/cdtA−/cdtB−*. The few strains from clade 4 were also very distant, except for adult strains 82 and 78 recovered in 2014; strains from this clade were non-toxigenic (*tcdA−/tcdB−/cdtA−*/*cdtB−*) or producing only one toxin (*tcdA−/tcdB+/cdtA−/cdtB−*). The single strain recovered from clade 5, children isolate 85, was very distant from all other isolates and presented the unusual pattern *tcdA−/tcdB+/cdtA+/cdtB*+. Thus, toxigenic strains *tcdA+/tcdB+/cdtA+/cdtB*+ from clade 2 have remained as the most prevalent strains in different adult and pediatric hospitals in Mexico City during the period 2014–2019, whereas strains from clades 1 and 3 varied genomically between hospitals and year of isolation.

## Discussion

Antimicrobial therapy is one of the most common risk factors for the development of CDI (Pépin et al., 2004). Consequently, drug resistance is a well-recognized problem among clinical isolates of *C. difficile* that has continued to increase in recent years (Mutai et al., 2021). In 2019, the Center for Disease Control and Prevention classified *C. difficile* as one of five urgent health threats and called for aggressive actions to counteract the significant risks associated with antimicrobial overuse (Mutai et al., 2021). Molecular epidemiology studies of CDI will provide better understand of virulence mechanisms in combination with resistance profiles to commonly used antibiotics so as to define links between distribution, prevalence, and associations with outbreaks that can be targeted for controlling and limiting health consequences. In this study, we used WGS with 94 *C. difficile* isolates to determine the phylogeny, cgMLST allelic profiles, toxin gene profile, and phenotypic and genotypic resistance to antibiotics in strains from pediatric and adult patients in Mexico. Consistent with other studies, the majority of toxigenic strains were *tcdA+/tcdB+/cdtA+/cdtB+* (RT027/ST1) (Aguayo et al., 2015), a toxin profile associated with many ribotypes including the globally distributed RT078 and RT 027.

In contrast to previous studies, we included in the analysis a group of pediatric patients to discover that the frequency of the *tcdA+/tcdB+/cdtA+/cdtB+* was significantly higher in adults compared to children (81% vs. 41.9%; *p* = 0.02). This observation highlights the need to understand the difference between the patient populations to understand how different toxin profiles impact the distribution and prevalence of CDI isolates linked to outbreaks. This agrees with the relatively few studies that reported infection in children with this toxins profile (Lukkarinen et al., 2009; Lees et al., 2020). Five isolates contained the combination of toxins *tcdA*−/*tcdB+/cdtA−/cdtB−* (four from adults and one from a child), which has been rarely reported in North America, although cases with this toxin combination genotype are increasing in Europe (Freeman et al., 2020) and Asia (Azimirad et al., 2018). Interestingly, this toxin grouping is commonly reported in pediatric cases in the Netherlands (12%) (Van Dorp et al., 2017). It should be noted that the toxin profiles were confirmed by genome sequence and thus not influenced by gene variants that could be missed by PCR. The use of orthogonal methods to find (PCR) and confirm (WGS) these results suggests that these observations are accurate and reflect the clinical situation in Mexico.

In pediatric patients, a significantly higher number of non-toxigenic isolates were found compared to those from adults (22.5% vs. 7.9%; *p* = 0.045), and this agrees with previous studies reporting that children are often colonized by non-toxigenic *C. difficile* isolates (Camorlinga-Ponce et al., 1987; Spigaglia and Barbanti, 2020). Of interest, isolates lacking one or more toxin genes were also more frequently isolated from children. This observation brings into question the role of these organisms in diarrheal episodes in pediatric cases.

MLST typing was determined from WGS of the 94 strains of *C. difficile* to reveal that 20 STs are circulating in hospitals within Mexico City. Differences in the diversity of STs in different geographical regions have been reported, for example, in one Asian region, 68 different STs were reported, whereas, in Colombia, 11 were found (Luo et al., 2019; Muñoz et al., 2019). According to the seven MLST housekeeping genes, a total of five distinct phylogenetic clades (clades 1, 2, 3, 4, and 5) have been described (Janezic and Rupnik, 2019). Four of these clades were identified in the isolates from this study (clade 1, 2, 4, and 5). *C. difficile* clade 2 (ST1) was the dominant MLST type in isolates from Mexico in children and adults. This MLST type (NAP1/ST1) is recognized to be hypervirulent and is responsible for outbreaks worldwide (Badilla-Lobo and Rodríguez, 2021). A previous report in Mexico identified *C. difficile* NAP1/027 hypervirulent in adults with nosocomial diarrhea (Camacho-Ortiz et al., 2015). In children, there has been a notable increase in CDI cases since 2002. In addition, reports suggest that they occur with disease increased severity (Noor and Krilov, 2018). However, there are few studies on infection with hypervirulent *C. difficile* NAP1/RT-027 in pediatric patients (Alvarez and Rathore, 2019). In this study, it was found that children in Mexico were frequently (48.3%) colonized with *C. difficile* clade 2 (ST1) strains. A previous report suggests that CDI is higher in children with cancer (Alvarez and Rathore, 2019) and, in our study, six of the 31 children were oncology patients. However, this study found that children with hospital-acquired diarrhea are often colonized with non-toxigenic strains (22.5%) or isolates that lack *cdt* toxin gene (*tcdA+/tcdB+/cdtA−/cdtB−*) (25.8%), a genotype profile that contrast with that observed in adults.

The epidemiology of CDI is highly dynamic with new strains continually emerging worldwide (Diniz et al., 2019). In contrast, studies in Mexico during the last 5 years suggest that the epidemiology of CDI has remained stable, with ST1 as the dominant MLST type (Camacho-Ortiz et al., 2015; Martínez-Meléndez et al., 2018). Clade 1 was the second most frequent MLST observed (28.2%) with 12 different STs and with different toxin profiles, most of them non-toxigenic (34%) or partially toxigenic (62%) and a very low fraction toxigenic (3.8%). It is well documented that a clade can be associated with more than one RT (Knight et al., 2015), and clade 1 is the most heterogeneous not only in terms of STs but also in its toxigenic profiles (Janezic and Rupnik, 2015), which agrees with our findings. Of note, 42% of the *C. difficile* isolates from children were of clade 1, whereas only 19% of those from adults belonged to this clade (*p* = 0.012), which suggest marked differences in the molecular epidemiology between adult and pediatric *C. difficile* strains.

Five strains were grouped in clade 4, and three of these strains were ST37, which is related to RT017/ST37 present in Europe, North America, and Argentina (Goorhuis et al., 2009; Imwattana et al., 2019). The presence of this clade is relevant because this ST has been associated with high levels of antibiotic resistance, which complicates CDI treatment and increases recurrence risk and the emergence of outbreaks.

One pediatric strain isolated in 2014 belonged to clade 5 (ST11), related to RT078, an emerging and hypervirulent strain reported in China, Japan, Australia, and Europe (Freeman et al., 2015; Li et al., 2019). This genotype was usually associated with infections in animals (Janezic and Rupnik, 2019), but it has now become a significant public health problem in humans. Finally, non-toxigenic strains in clade 1 (STs 15 and 26) have been reported only in Oxfordshire, United Kingdom (Dingle et al., 2011), but, now, we documented its presence in Mexico.

We found that all the isolates were susceptible to metronidazole and vancomycin, which is consistent with the use of these antibiotics as the first-line for treatment of CDI (Spigaglia, 2016; Peng et al., 2017; Mutai et al., 2021). A low prevalence of resistance to metronidazole and vancomycin has been reported in some countries (Goudarzi et al., 2013; Muñoz et al., 2019), although reports of treatment failure with metronidazole are increasing (Chahine, 2018). Resistance to vancomycin has been documented in strains from Iran, Israel, Italy, and Spain (Peng et al., 2017), and resistance as high as 58% was found in strains from Brazil (Fraga et al., 2016).

We found a high proportion of resistance to fluoroquinolone in both children and adult isolates, except for levofloxacin that was significantly lower in isolates from children (see Table 1). A lower resistance to moxifloxacin in children than in adult strains has also been reported (Kociolek et al., 2016). The high resistance of *C. difficile* to fluoroquinolones is possibly related to the high proportion of NAP1/RT-027 strains found in our isolates, because high resistance to fluoroquinolones has been reported worldwide in this genotype (Miller et al., 2010; Kociolek et al., 2016). In agreement with the high prevalence of resistance to fluoroquinolones, we found substitutions in the *gyrA* or *gyrB* genes in most of the isolates (69%), although with a higher frequency in adults than in pediatric isolates. Among the 88 strains resistant to moxifloxacin 65 (73.8%) contained a SNP in *gyrA* or *gyrB* presenting the SNPs Thr82Ile, Leu406Ile, and Asp468Asn that are the same observed in most resistant *C. difficile* strains worldwide (Spigaglia et al., 2010; Mac Aogáin et al., 2015). Interestingly, these three SNPs had a low concordance with the phenotype, whereas all other SNPs in both *gyrA* and *gyrB* showed no agreement.

Resistance to erythromycin and clindamycin is the most common phenotype among *C. difficile* strains isolated in Europe (Wasels et al., 2015) and the *erm(B*) gene, the most common determinant of resistance to the macrolide–lincosamide–streptogramin (MLS_*B*_) family. In this study, we identified *erm(B)* in 60 of 83 (72.2%) *C. difficile* isolates that were resistant to clindamycin. In contrast, Ackermann et al. (2003) found that 51% of MLS_*B*_-resistant C. *difficile* strains were negative for *ermB* and suggested that resistance could be the result of mutations in the target sequences in the 23S rRNA gene.

Analysis of the sensitivity of *C. difficile* to rifamycin showed a higher number of resistant strains in adults (85%) than in children (64.5%) (*p* = 0.018). Reports in other regions have found lower resistance rates; a study in the United States reported resistant to rifampicin in 1.6% of pediatric and 6.7% of adult isolates (Kociolek et al., 2016), whereas a European study found 13.4% of resistance for this antibiotic (Freeman et al., 2015). Resistance has been associated with point mutations in the *rpoB* gene (O’Connor et al., 2008), and we detected mutations in the *rpoB* (Arg505Lys, Ile548Met, and Asp1232Glu) and *rpoC* (Ile833Leu) genes that showed a moderate agreement with the phenotype.

Resistance to linezolid has been occasionally described in clinical isolates of *C. difficile* (Marín et al., 2015), and it has been associated with *cfr* genes (Stojković et al., 2020). A previous report identified the *cfr* gene as possible mechanism of resistance to linezolid in seven of nine resistant strains (Marín et al., 2015), and, recently, *cfr*E was identified in a Mexican strain resistant to linezolid (Stojković et al., 2020). In this study, we described the presence of *cfrE* gene in linezolid resistant ST1 strains from children and adults and confirmed its dissemination among strains in Mexico (Stojković et al., 2020).

Sequencing of *C. difficile* genome offers an opportunity to identify genes and mutations associates with resistance to antibiotics and allows prediction of the antibiotic resistance phenotype, but validation of the phenotype–genotype correlation in each region is needed. We found no or low phenotype–genotype correlation for most of the studied mutations related to quinolones and meropenem resistance. However, a moderate agreement was found for rifampicin and an excellent agreement for fusidic acid (Table 2), suggesting the utility of these markers in our community. The *tet*M gene was a poor predictor for tetracycline susceptibility phenotype, similar to results reported by Xu et al. (2021).

We analyzed the genomes of our isolates with virtual hybridization and cgMLST using the EnteroBase platform for better reference with other studies. Genome analyses allowed a better discrimination of strains and showed that closely related strains belonging to clade 2, toxigenic, and with high antibiotic resistance have been predominant in children and adult isolates from different hospital during the last 5 years. The analysis also showed the high genome diversity within strains from clades 1 and 4 with no clustering across years of isolation or hospitals as observed with clade 2 isolates. Thus, strains with variable toxins profile or antibiotic resistance and high genome diversity are continuously emerging and should be regularly monitored.

In summary, in the current study, we reported the molecular epidemiology of *C. difficile* strains based on a WGS analysis of genotypes, virulence genes, and antimicrobial resistance in hospitals of Mexico. An important strength of this study is the inclusion of *C. difficile* isolates from pediatric and adult patients. Our data show a high prevalence of NAP1/ST1 infection and significant diversity in ST and in toxins profiles, with differences between adult and pediatric isolates. Whereas all *C. difficile* strains were sensitive to metronidazole and vancomycin, a high prevalence of multi-resistance to fluoroquinolones, clindamycin, rifampicin, linezolid, and meropenem was found. The genotype and phenotype agreement were low for most antimicrobials, except for quinolones and fusidic acid. Effective antimicrobial administration and infection control programs are needed to prevent and contain the spread of multidrug resistant and potentially epidemic strains of *C. difficile*.

## Data Availability Statement

The datasets presented in this study can be found in online repositories. The names of the repository/repositories and accession number(s) can be found in the article/Supplementary Material.

## Ethics Statement

The studies involving human participants were reviewed and approved by the Ethics Committee Comite Nacional de Investigación Cientifica Numero R-2015-785-089. Written informed consent to participate in this study was provided by the participants’ legal guardian/next of kin.

## Author Contributions

MC-P and JT designed and coordinated the study and wrote the manuscript. EA-Z performed the isolation, antimicrobial susceptibility, analysis of results, and wrote the manuscript. NO-O selected patients. AG-D participated in statistical analysis. GA-O and VB-B contributed to revising the article and isolation of *C. difficile* strains. RT participated in the bioinformatic analysis. BW performed the sequencing of the genome. All authors contributed to the article and approved the submitted version.

## Conflict of Interest

The authors declare that the research was conducted in the absence of any commercial or financial relationships that could be construed as a potential conflict of interest.

## Publisher’s Note

All claims expressed in this article are solely those of the authors and do not necessarily represent those of their affiliated organizations, or those of the publisher, the editors and the reviewers. Any product that may be evaluated in this article, or claim that may be made by its manufacturer, is not guaranteed or endorsed by the publisher.

## Footnotes

1
http://www.eucast.org

2
https://github.com/tseemann/shovill

3
http://bioinf.spbau.ru/quast

4
https://github.com/tseemann/prokka

5
https://cge.cbs.dtu.dk/services/ResFinder/

6
https://enterobase.warwick.ac.uk

7
http://github.com/tseemann/mlst

8
https://card.mcmaster.ca

9
https://github.com/tsee-mann/abricate

10
https://github.com/tseemann/snippy

11
http://biomedbiotec.encb.ipn.mx/VAMPhyRE

12
http://www.sergas.es/Saude-publica/EPIDAT
