# Supplementary material for: Molecular Epidemiology and Antimicrobial Resistance of Clostridioides difficile in Hospitalized Patients From Mexico
Source: Front Microbiol. 2022 Mar 10;12:787451. doi: 10.3389/fmicb.2021.787451 (PMC8960119; doi:10.3389/fmicb.2021.787451)
Supplement: Supplementary file 1 [file Data_Sheet_1.docx]

| **ID** | **Adult/Children** | **Age** | **Sex** | **NCBI Number** | **Short-reads archive**  **Number** |
| --- | --- | --- | --- | --- | --- |
| 16 | Adult | 72 | F | SAMN21923160 | SRR17242271 |
| 19 | Adult | 60 | F | SAMN21923161 | SRR17242270 |
| 24 | Adult | 70 | F | SAMN21923162 | SRR17242259 |
| 25 | Adult | 70 | F | SAMN21923163 | SRR17242248 |
| 29 | Adult | 69 | M | SAMN21923164 | SRR17242237 |
| 36 | Adult | 60 | M | SAMN21923165 | SRR17242226 |
| 37 | Adult | 24 | F | SAMN21923166 | SRR17242215 |
| 38 | Adult | 50 | M | SAMN21923167 | SRR17242204 |
| 41 | Children | 12 | M | SAMN21923168 | SRR17242193 |
| 47 | Adult | 58 | F | SAMN21923169 | SRR17242182 |
| 61 | Adult | 80 | F | SAMN21923170 | SRR17242269 |
| 66 | Adult | 66 | F | SAMN21923171 | SRR17242268 |
| 78 | Adult | 60 | F | SAMN21923172 | SRR17242267 |
| 82 | Adult | 57 | F | SAMN21923173 | SRR17242266 |
| 85 | Children | 0.7 | F | SAMN21923174 | SRR17242265 |
| 91 | Adult | 81 | F | SAMN21923175 | SRR17242264 |
| 95 | Children | 13 | M | SAMN21923176 | SRR17242263 |
| 103 | Adult | 81 | M | SAMN21923177 | SRR17242262 |
| 107 | Adult | 80 | f | SAMN21923178 | SRR17242261 |
| 110 | Children | 1 | F | SAMN21923179 | SRR17242260 |
| 111 | Adult | 66 | F | SAMN21923180 | SRR17242258 |
| 128 | Adult | 48 | M | SAMN21923181 | SRR17242257 |
| 129 | Adult | 70 | M | SAMN21923182 | SRR17242256 |
| 138 | Adult | 46 | F | SAMN21923183 | SRR17242255 |
| 140 | Children | 3 | F | SAMN21923184 | SRR17242254 |
| 144 | Adult | 60 | F | SAMN21923185 | SRR17242253 |
| 146 | Adult | 67 | F | SAMN21923186 | SRR17242252 |
| 148 | Children | 13 | F | SAMN21923187 | SRR17242251 |
| 160 | Adult | 65 | F | SAMN21923188 | SRR17242250 |
| 166 | Adult | 37 | M | SAMN21923189 | SRR17242249 |
| 167 | Adult | 51 | M | SAMN21923190 | SRR17242247 |
| 171 | Adult | 75 | F | SAMN21923191 | SRR17242246 |
| 174 | Adult | 49 | F | SAMN21923192 | SRR17242245 |
| 184 | Children | 2 | M | SAMN21923193 | SRR17242244 |
| 186 | Adult | 65 | F | SAMN21923194 | SRR17242243 |
| 197 | Adult | 26 | F | SAMN21923196 | SRR17242242 |
| 205 | Children | 2 | F | SAMN21923197 | SRR17242241 |
| 232 | Adult | 55 | F | SAMN21923198 | SRR17242240 |
| 253 | Children | 3 | F | SAMN21923199 | SRR17242239 |
| 267 | Children | 15 | M | SAMN21923200 | SRR17242238 |
| 268 | Children | 15 | M | SAMN21923201 | SRR17242236 |
| 271 | Children | 0.7 | F | SAMN21923202 | SRR17242235 |
| 275 | Children | 15 | M | SAMN21923203 | SRR17242234 |
| 281 | Adult | 66 | F | SAMN21923204 | SRR17242233 |
| 285 | Children | 14 | M | SAMN21923205 | SRR17242232 |
| 291 | Children | 6 | M | SAMN21923206 | SRR17242231 |
| 295 | Adult | 76 | F | SAMN21923207 | SRR17242230 |
| 299 | Adult | 51 | F | SAMN21923208 | SRR17242229 |
| 300 | Adult | 63 | M | SAMN21923209 | SRR17247447 |
| 322 | Adult | 72 | F | SAMN21923210 | SRR17242228 |
| 330 | Children | 15 | F | SAMN21923211 | SRR17242227 |
| 336 | Adult | 35 | F | SAMN21923212 | SRR17242225 |
| 361 | Adult | 66 | M | SAMN21923213 | SRR17242224 |
| 365 | Adult | 82 | M | SAMN21923214 | SRR17242223 |
| 367 | Children | 1 | M | SAMN21923215 | SRR17242222 |
| 375 | Children | 14 | M | SAMN21923216 | SRR17242221 |
| 379 | Children | 4 | M | SAMN21923217 | SRR17242220 |
| 391 | Children | 13 | F | SAMN21923218 | SRR17242219 |
| 392 | Children | 5 | M | SAMN21923219 | SRR17242218 |
| 395 | Adult | 66 | F | SAMN21923220 | SRR17242217 |
| 424 | Children | 8 | M | SAMN21923221 | SRR17242216 |
| 429 | Children | 2 | M | SAMN21923222 | SRR17242214 |
| 432 | Adult | 40 | F | SAMN21923223 | SRR17242213 |
| 457 | Children | 2 | M | SAMN21923224 | SRR17242212 |
| 458 | Adult | 61 | F | SAMN21923225 | SRR17242211 |
| 479 | Children | 12 | F | SAMN21923226 | SRR17242210 |
| 492 | Children | 10 | M | SAMN21923227 | SRR17242209 |
| 501 | Children | 2 | M | SAMN21923228 | SRR17242208 |
| 536 | Adult | 65 | F | SAMN21923229 | SRR17242207 |
| 543 | Children | 18 | F | SAMN21923230 | SRR17242206 |
| 582 | Adult | 66 | M | SAMN21923231 | SRR17242205 |
| 591 | Children | 0.4 | M | SAMN21923232 | SRR17242203 |
| 597 | Adult | 43 | F | SAMN21923233 | SRR17242202 |
| 632 | Children | 10 | F | SAMN21923234 | SRR17242201 |
| 635 | Children | 3 | M | SAMN21923235 | SRR17242200 |
| 636 | Children | 12 | F | SAMN21923236 | SRR17242199 |
| 653 | Adult | 82 | M | SAMN21923237 | SRR17242198 |
| 656 | Adult | 80 | M | SAMN21923238 | SRR17242197 |
| 664 | Adult | 83 | F | SAMN21923239 | SRR17242196 |
| C03 | Adult | NA | M | SAMN21923240 | SRR17242195 |
| C05 | Adult | NA | M | SAMN21923246 | SRR17242194 |
| C11 | Adult | NA | M | SAMN21923241 | SRR17242192 |
| C13 | Adult | NA | M | SAMN21923242 | SRR17242191 |
| C16 | Adult | NA | M | SAMN21923243 | SRR17242190 |
| C18 | Adult | NA | M | SAMN21923244 | SRR17242189 |
| C20 | Adult | NA | M | SAMN21923245 | SRR17242188 |
| MS04 | Adult | 57 | F | SAMN21923247 | SRR17242187 |
| MS08 | Adult | 42 | M | SAMN21923248 | SRR17242186 |
| MS10 | Adult | 59 | F | SAMN21923249 | SRR17242185 |
| MS11 | Adult | 25 | F | SAMN21923250 | SRR17242184 |
| MS12 | Adult | 76 | F | SAMN21923251 | SRR17242183 |
| MS14 | Adult | 32 | M | SAMN21923252 | SRR17242181 |
| MS15 | Adult | 23 | M | SAMN21923253 | SRR17242180 |
